# Pediatric Healthcare Needs and Barriers Self-Reported by a Rural Hispanic Community

**DOI:** 10.7759/cureus.11999

**Published:** 2020-12-09

**Authors:** Juan J Leon, Kourtney K Guthrie, Carolina Rueda, Diomel De la Cruz, Diana Montoya-Williams

**Affiliations:** 1 Pediatrics, Herbert Wertheim College of Engineering, Gainesville, USA; 2 Pediatric- Critical Care, University of Florida Health Shands Hospital, Gainesville, USA; 3 Miscellaneous, The University of Florida College of Liberal Arts and Sciences, Gainesville, USA; 4 Pediatrics - Neonatology, University of Florida Health Shands Hospital, Gainesville, USA; 5 Pediatrics • Neonatology, Children's Hospital of Philadelphia, Philadelphia, USA

**Keywords:** immigrant, latino, health disparities, underserved, children

## Abstract

Purpose

Hispanic immigrants in rural communities can be a hard-to-reach population with many unmet medical needs that have yet to be properly identified. This is particularly true for rural immigrant children. This study focused on documenting self-reported healthcare barriers among this isolated population to identify methods for reducing health disparities among this vulnerable population.

Methods

Participants at recurrent mobile health fairs were anonymously surveyed from June 2016 to January 2018. Differences between the US and foreign-born Hispanic participants were investigated in bivariate analyses.

Findings

We received 35 completed surveys. The majority (79.3%) of participants reported household incomes below the federal poverty line for a family of four. Only 4.5% of foreign-born children were insured, compared with 77% of US-born children (p<0.001). Greater than 85% of foreign-born and 100% of US-born children were fully vaccinated, but half of the participants were seeking preventative care. Most patients identified insurance and cost as the most significant barriers to healthcare.

Conclusions

Access to insurance was the largest barrier identified by this population, with a significant difference between foreign and US-born children. This gap is further compounded by many responders living below the federal poverty line, limiting their ability to pay for the growing costs of uninsured treatment. This study also indicates this Hispanic community's prioritized desire for access to preventative healthcare and high uptake of childhood immunizations.

## Introduction

Hispanics are currently the largest minority population in the United States [1]. A clear link has previously been drawn between immigrant status and limited access to healthcare, with English proficiency and lack of insurance is commonly cited barriers [2,3]. Immigrant children are also often limited in their access to healthcare, leading to disparities between foreign and US-born children [4]. While healthcare access for adult immigrant populations has been extensively explored, there is less information regarding the health needs of immigrant children, particularly in rural communities [4]. This is concerning given that there is a growing trend of rural relocation primarily amongst Hispanic immigrant groups [5] and that young Hispanics are more prone to being uninsured if they live in rural areas [5]. While public policy solutions involving free health clinics have been previously utilized to address these issues among Hispanic communities [4], more research is required to identify the specific needs of rural Hispanic patients of all ages and their responses to such public health projects [2,3,6].

Prior studies have suggested that local hospital-based safety net programs are not enough for most Hispanic immigrants, only partially meeting healthcare needs [7]. For instance, non-emergency care for older, nonworking Latino adults remains a significant unmet necessity in the American Midwest [8]. This hints at the growing need to identify not only population-specific but also age-specific requirements of rural Hispanic immigrants. Similarly, a lack of health insurance has been directly tied to reduced healthcare access, leading to the loss of continued healthcare services, decreased clinic visits, and reduced satisfaction with individual ability to obtain care [9].

Previous efforts to gather data from attendees of community health events have provided critical insights into the needs of a given community, highlighting the possibility of discovering crucial information that might significantly impact that population [4]. Also, trends have been identified in Hispanic populations, pointing towards a greater tendency to seek healthcare when informed of resource availability [10]. This underscores the importance of identifying Hispanic families' medical needs as they may show greater uptake of healthcare recommendations [10]. Given that existing racial/ethnic health disparities are exacerbated when rural community needs are left unaddressed [11], we aimed to capture the health needs of Hispanic patients in a rural farm-working community. Our overarching goals were to assess better the needs of the community our group was serving through advocacy-based medical outreach, and, more globally, to highlight a strategy that may help combat the disparities faced by rural immigrant children.

## Materials and methods

This study was designed as a prospective survey. Patient information was obtained via a voluntary, anonymous survey offered to families who attended a local quarterly pediatric-oriented health fair and clinic organized jointly by various community stakeholders including a local nonprofit, a faith-based organization, and an academic medical institution. Health fairs consisted of a mobile clinic staffed by local healthcare providers as well as public health education workshops for families and tabling of local social service programs to increase community awareness of available resources. The health fairs were founded by paediatricians looking to bring pediatric care to this community; however, patients of all ages were seen at the clinics. Fairs were advertised via flyers created by the nonprofit organization, the dissemination of which was coordinated through local community leaders and partner community immigrant health advocacy groups. Flyers were also sent out to local schools after approval from the area's public school board.

The survey was aimed at identifying the general profile and health needs of pediatric patients being seen in the clinic. It consisted of 13 items that asked about family socio-demographics, including insurance status, the existence of an established medical home for the child, perceived barriers to healthcare, and desired services. The survey was written in English and had a Flesch-Kincaid reading grade level of 0. It was translated into Spanish and back-translated to confirm accuracy by a professional interpreter company. Participants could choose to answer the survey in their preferred language.

Our study period overlapped with a period during which anti-immigrant rhetoric was rising, and immigration policies were becoming more aggressive and punitive. Our community partners provided us with insight regarding the rising fear and anxiety in our immigrant community as a result of these cultural and policy shifts. As such, we chose not to collect documentation status in this study to mitigate the risk of exacerbating these concerns and impacting families' willingness to receive care and resources at our health fairs. However, we did collect information on the length of time in the US and insurance status, two variables that have been used by other studies as a proxy for legal status [12].

Survey data collection took place throughout five health fairs, held between June 2016 and January 2018. The survey was offered to clinic participants as they checked in by non-research personnel. If the patient who checked in was under 18, the survey was offered to the parent or caregiver present with the child. Vaccination status was determined through caregiver self-report.

Analyses consisted of descriptive statistics of respondents' answers. Also, bivariate analyses were conducted to assess for differences within the survey cohort by foreign-born status and presence of insurance using either Chi-Square or Fisher's exact tests. As this was an exploratory community needs assessment using a previously nonexistent outreach event to recruit participants, it was not possible to know beforehand how many potential participants would present to the health fair. Thus, power calculations for sample size were not performed. All analyses were conducted with IBM(c) Statistical Package for the Social Sciences (SPSS) Version 25. The local academic Institutional Review Board approved this study.

## Results

Out of the 70 total patients seen by the clinic during the study period, 35 survey responses were recorded. Surveys completed about participants 18 years old or younger made up the bulk of responses (85%). Although surveys were filled out by adult caregivers versus the pediatric patients themselves, responses will describe the subject of the survey (i.e. the child being seen in the clinic).

The majority of our participants were foreign-born (63%), with 41% of these being from South America and 24% being from Central America. A third of participants chose not to indicate where they were from. Just under half (45.5%) of self-identified immigrant participants reported being in the US for less than one year, and 45.5% reported being in the country for one to five years. Table 1 summarizes the rest of the demographic and medical characteristics of our survey participants and compares the responses of US vs foreign-born participants. The majority of participants were younger than 18, male, and living in families with annual income between $10,000-$20,000. The highest level of parental education was split between less than high school and completed college. Of the patients surveyed, only 31.4% were insured, and 37.1% had a current primary care provider (PCP), with the majority reporting no insurance or access to a PCP. Greater than 85% of foreign-born patients were up to date on vaccines, while 100% of US-born children were fully immunized per caregiver report. Greater than 95% of our respondents reported living within 30 minutes of the clinic location, indicating we were serving a very local community.

When comparing foreign-born to US-born patients, insurance rates among foreign-born children were significantly lower than among US-born children (4.5% vs 77% respectively, p<0.001). Similarly, far fewer foreign-born children had access to a PCP compared to US-born children (18.2% vs 69.2%, p=0.004) (Table 1). Most patients presented to the clinic for a preventative well visit. They identified insurance and cost as the most significant barriers to care, with a lack of knowledge of the healthcare system as the third most common barrier. Primary care and dental care arose as the most significant medical needs; vaccines were the next most cited need (12.5%), despite high rates of fully vaccinated patients (Table 2).

**Table 1:** Demographic Characteristics of Survey Participants *Obtained via Fisher’s exact tests vs. Chi Square tests due to some cell sizes being <5.

Demographic	Entire Cohort (%, N=35)	Foreign Born (%)		US Born (%)		P-value		
Age								
< 1	5.7	4.5		7.7		0.953		
2-5 years	17.1	18.2		15.4				
6-10 years	37.1	36.4		38.5				
11-17	28.6	31.8		23.1				
>18	11.4	9.1		15.4				
Gender (% female)	45.7	45.5		46.2		0.968		
Familial Annual Income								
Less than $10000	24.1	31.6		10.0				
$10-20,000	55.2	68.4		30.0				
$20-40,000	20.7	0		100				
Did not answer	20.7							
Parental Education								
None	2.9	4.5		0		0.625		
Less than High School	34.3	18.2		7.7				
High School	20.0	13.6		30.8				
College	42.9	45.5		38.5				
Live within 30 minutes of health fair	97.0	95.5		100.0		0.538		
Insured	31.4	4.5		77.0		<0.001*		
Have a Primary Care Provider	37.1	18.2		69.2		0.004*		
Up to date on vaccines	91.0	85.7		100.0		0.284*		

**Table 2:** Participants’ Self-Reported Needs & Barriers to Care

	N (%)	Foreign-Born %	US-Born %
Reasons for Presenting to Mobile Clinic			
Well visit	17 (50%)	94.1%	5.9%
School/sports physical	13 (38.2%)	69.2%	30.8%
Dental care	10 (29.4%)	50.0%	50.0%
Vaccines	6 (17.8%)	100%	0
Sick visit	2 (5.9%)	50.0%	50.0%
Other	3 (8.9%)	33.3%	66.7%
Barriers to Healthcare			
Lack of Insurance	19 (65.6%)	89.5%	10.5%
Cost	17 (58.6%)	82.4%	17.7%
Knowledge of System	11 (37.9%)	81.8%	18.2%
Documentation Concerns	9 (31.0%)	77.8%	22.2%
Transportation	2 (6.9%)	50%	50%
Self-Identified General Needs			
Primary Care	21 (60.0%)	76.2%	23.8%
Dental Care	12 (37.5%)	50%	50%
Vaccines	4 (12.5%)	100%	0
Specialty Care	1 (3.1%)	100%	0
Nutrition Education	1 (3.13%)	100%	0
Other	1 (3.13%)	0	100%

## Discussion

In this assessment of the pediatric healthcare needs and barriers self-reported by a group of Hispanic immigrant families in a rural farm-working community, we found health insurance was one of the major barriers to healthcare access, with most survey participants reporting uninsured status. This finding provides further evidence for the ongoing unmet need for insurance coverage among immigrant families. More work is critically needed to understand better and address the overall lack of insurance in our study population, and particularly among US-born children. It is possible, for instance, that our findings indicate early evidence of a "chilling effect" whereby families were not seeking services their children might qualify for due to immigration status-related fears [13]. However, this requires better exploration in future studies.

Our survey also indicated difficulty understanding the US healthcare system, which may impede families' ability to access resources that are available for their children. Primary care was the most requested type of healthcare, reinforcing the necessity of low-cost preventative health strategies within rural immigrant communities who may live far from safety-net health departments and large medical centres in urban areas. The request from this community for dental care as the second most sought-after type of care is in line with the findings of other groups who have worked with similarly vulnerable undocumented or migrant immigrant communities: access to dental care is a significant unmet need [14,15].

We believe an important finding from this work is that this Hispanic community appeared to prioritize access to preventative healthcare. Despite insurance barriers and work obligations, child vaccination rates were high, even among foreign-born participants. This speaks to a high level of trust and acceptance of vaccinations among Hispanic immigrants, as has been previously documented among Hispanic parents for other immunizations, like the HPV vaccine [10]. Given the global public health threat that vaccine hesitancy poses [16] and consequently, the waning herd immunity in diseases like measles, mumps, and other vaccine-preventable illnesses [17], decreasing barriers to immunizations among Hispanic immigrant families may be an efficient and cost-effective public health strategy. Also, given that patient trust in providers plays a role in access to and utilization of medical care [18], our indirect evidence of trust among Hispanic patients should be further explored and optimized to help improve health disparities among Hispanic families.

Another important, though unsurprising, the finding of our work was that US-born participants were substantially more likely to have insurance than their foreign-born counterparts highlighting a massive gap in coverage for rural, foreign-born Hispanic immigrants. Also, foreign-born participants reported extreme poverty, with family incomes not only far below the federal poverty line for a family of four but also nearly half the median income reported by farm-workers in the United States in the most recent National Agricultural Workers Survey (NAWS) [19]. These findings indicate that primary healthcare likely imposes significant if not unsurmountable financial strain, particularly for foreign-born individuals as compared with their US-born counterparts. Uninsured immigrant families in our area have very few options for affordable health care; most of these options employ a fee-for-service model with a sliding scale and require a paycheck. This highlights why such families rely on safety-net programs such as our mobile clinic and why national studies of healthcare expenditures show that immigrants are more likely to receive uncompensated care, either as charity care or as emergency care that goes unpaid-for [20]. Expanding insurance coverage among immigrants regardless of country of origin or citizenship status would not only assist communities (such as ours) access regular preventative healthcare, but prior research indicates it would be cost-effective [20].

Limitations of this study include a low survey response rate from a small original sampling population. Also, although the survey was designed to be as accessible and non-threatening as possible, survey responses may have been impacted by the fear of providing personal information or literacy issues. Despite this, we believe the strength of this study is its contribution to our understanding of needs that are often difficult to assess from this hard-to-reach population. Furthermore, our findings continue to underscore the fact that Hispanic immigrant families readily seek and welcome preventative healthcare, mostly if it is made available via a non-threatening venue with the visible support of trusted local stakeholders. Our community-university partnership continues to assist us in creating such a space; thus, we believe such partnerships are essential when working with this particularly vulnerable community. Future planned work includes an impact evaluation of our approach for quality improvement purposes.

## Conclusions

In this survey of families seeking care at free health fairs in rural Florida, we found low rates of insurance coverage and access to a medical home among immigrant families, even among US-born children within immigrant families. We also found high rates of child vaccination despite insurance gaps. Our work highlights the need to better explore strategies to increase insurance coverage among eligible Hispanic immigrant children, remove other barriers to preventative medical and dental care, and explore the role of trust between healthcare providers and Hispanic parents on Hispanic children’s health outcomes.
